# Will urban scale affect health services inequity? The empirical evidence from cities in China

**DOI:** 10.3389/fpubh.2024.1330921

**Published:** 2024-07-08

**Authors:** Hongchuan Wang, Kaibo Xu, Handong Fang, Hui Lin, Huatang Zeng

**Affiliations:** ^1^School of Public Policy & Management, Tsinghua University, Beijing, China; ^2^School of Economics and Management, Tsinghua University, Beijing, China; ^3^School of Public Health, Li Ka Shing Faculty of Medicine, The University of Hong Kong, Pokfulam, Hong Kong SAR, China; ^4^Shenzhen Health Development Research and Data Management Center, Shenzhen, Guangdong, China; ^5^Vanke School of Public Health, Tsinghua University, Beijing, China

**Keywords:** city shrinkage, health service, equity, resource allocation, population change

## Abstract

**Background:**

The equity of public resources triggered by city shrinkage is a global challenge. Significantly, the impact of city shrinkage on the allocation of health service resources needs to be better understood. This study explores the impact of population change on government investment and health service delivery in shrinking cities.

**Data and method:**

Using data from China’s Urban Statistical Yearbook (2010–2020), we employ regression discontinuity (RD) and fixed-effect models to examine the causal relationship between city shrinkage and health service provision.

**Result:**

Shrinking cities show significant disparities in health resources, particularly in bed numbers (−1,167.58, *p* < 0.05) and doctor availability (−538.54, *p* < 0.05). Economic development (*p* < 0.01) and financial autonomy (*p* < 0.01) influence hospital bed distribution. Investments in public services (primary schools and teachers, *p* < 0.01) affect health resource delivery. Robustness tests support our results.

**Conclusion:**

This study reveals how city shrinkage disrupts health service provision and equity, establishing a causal relationship between city shrinkage/expansion and health resource allocation, emphasizing the imbalance caused by urban population changes. City expansion intensifies competition for health resources, while shrinking cities struggle to provide adequate resources due to government reluctance. Policymakers should adapt health resource allocation strategies to meet patient demands in changing urban landscapes.

## 1 Introduction

The demographic change poses public service challenges, as various factors associated with urbanization, such as demographic changes, socio-economic disparities (1), and regional development imbalances, may significantly impact and require careful allocation of health resources (2, 3). Several studies indicated that population decline indirectly results in the unsustainable maintenance and operation of urban infrastructure, ultimately reducing the quality of urban amenities (4). Modern cities compete for health services and city development, providing opportunities for health advances in city development. If competition aims to increase productivity, it may exacerbate the unequal distribution of health service resources between cities, and the country or region will face significant health service disparities (5). In addition, with the acceleration of urbanization, the demand for residents’ medical and health services has been released to a greater extent (6). However, the difference in urbanization may also lead to a gap in healthcare services.

Some studies have focused on the relationship between city population and the demand for and delivery of public service resources. Areas with high urbanization and population levels tend to have poorer ecological environments, negatively impacting the population’s health (7). Patient mobility leads to changes in the spatial economy of health services, resulting in the imbalanced allocation of medical service resources (8). Therefore, population dynamics, including population growth and loss, can impact public service delivery in a city. Population loss is considered an essential determinant of city shrinkage, a significant issue in city development referring to a decrease in city population or reduction in population density. Several factors contribute to city shrinkage, including suburbanization, disasters, an aging population, low fertility rates, etc. (9, 10).

Population decline can lead to reduced fiscal revenue, idle and wasted public service facilities, and impact the accessibility of services for residents. Many developed countries face this problem. For example, South Korea has experienced increased housing vacancy rates in city centers due to city shrinkage (11). In 1975, New York City faced a fiscal crisis attributed to population decline, economic factors, and political challenges; however, the government’s fiscal cuts primarily affected public services in impoverished areas of the city, including fire facilities, leading to severe fires in slum areas, exacerbating the plight of the underprivileged population (12). Researchers found that this policy resulted in a continuous deterioration of health conditions among vulnerable groups over the next 20 years (13). In many cases, city shrinkage affects residents in different areas differently, as various service reductions, including in health services, affect resource allocation fairness. However, policies can be implemented to improve the situation and ensure that those with greater needs can access a greater share of health services (14).

The issue of equity between different areas is difficult to deal with, but there are ways to mitigate the problem of city shrinkage so that it does not affect all groups. Some cities have counteracted this trend through industrial transformation and upgrading (15). Some have increased public services or health expenditures to improve health equity (16, 17). Health resource allocation is essential to effective city management and meeting the challenges of city shrinkage (18). Some studies suggested that urban–rural medical disparities be balanced to improve service quality and reduce cross-regional health services (19). Therefore, there is a need to rationalize resource allocation with a city’s population dynamics (20).

Our study explores the impact of city shrinkage on health service provision and contributes to the field in several new ways. First, it is concerned with allocating resources for health services in the context of shrinking cities. Despite its importance in city development, this area has not yet received much research attention. Second, it examines the role of the population in government decisions about health services delivery. Shrinkage cities with declining populations may also be urbanizing (21). Moreover, our research focuses on cities in developing countries with economic and social challenges affecting health service allocation.

## 2 Literature review

### 2.1 Utilization and distribution of health resources

The overall health system is unstructured, complex, and pluralistic, lacks effective coordination and governance, and has many actors with different vested interests (22). Due to these characteristics, there are many regions in the world facing the problem of shortage or waste of health resources, which essentially reflects the imbalance of health resources distribution (23). Global health resources are usually provided by different institutions, such as public hospitals, private hospitals, profit, and non-profit organizations. Public hospitals are the main providers of health resources in most countries, and their supply is more reflected in relatively unprofitable services, which are the most needed public health resources for ordinary people (24). Therefore, the resources of public health services can reflect the basic health level of a place. The imbalance in the distribution of public health resources is mainly caused by the political tradition and concept of the country, which leads to various inequalities through the transmission mechanism of the labor market and the national redistribution system (including the proportion of government expenditure on public health and services) (25). This inequality further affects the health status of individuals, such as differences in infant mortality and life expectancy (26). Now, with the improvement of economic development, people’s demand for health services is increasing, and the public health resources in many developing countries are facing great challenges in this situation (27, 28). In the face of serious challenges, many developing countries are more prone to the distribution of health resources, and this lack of access to health services has exacerbated the poverty situation of the lower class, leading to the widening trend of health inequality (29). Many of the most effective pro-poor health interventions remain underutilized, while less cost-effective interventions are funded. In these developing countries, the allocation of health resources is even more unequal (30).

The unequal distribution of health resources is also one of the negative externalities of some institutional reforms, such as fiscal decentralization, which reduces the government’s ability to redistribute income, and with reduced transfer payments and tighter local budget constraints, governments in poor areas must cut spending on social development (31). Secondly, increasing market competition has also severely affected state-owned enterprises, as it is difficult for them to fulfill the dual tasks of maximizing profits and providing welfare at the same time. As a result, state-owned enterprises in many countries have embarked on a process of privatization or mixed ownership reform (32). Others have had to lay off workers and cut benefits (33). So, their original role as providers of social welfare has thus been weakened. At the same time, due to the scale effect and agglomeration effect, both state-owned and private enterprises are concentrated in big cities, the government income in big cities is increased, and the allocation of medical resources is particularly generous (34). For some developing countries (such as China, India, etc.), the density of hospitals and the number of high-end service equipment in some big cities have reached the level of Western developed countries, but small cities or other areas have returned to the state of shortage of doctors and drugs, but deepened the inequality of public health resources (35, 36).

There are many existing factors leading to the unequal allocation of health resources. Specifically, the overall investment is limited, this means that when resources are allocated, regions are faced with limited funds for the construction of medical facilities, training of medical personnel, and implementation of public health programs. This lack of overall expenditure directly affects the soundness and popularization of the health service system, making the allocation of public health resources more difficult in some remote areas, especially in poor areas (37). On the other hand, high levels of public health investment do not necessarily guarantee full equity, if the cost of health care is high, then some socially and economically disadvantaged groups cannot afford the high cost of health care. This has led to certain socioeconomic disparities in the utilization of health services, making it possible for some people to lack access to timely and quality medical care (38). In addition, the non-profit nature of public hospitals and the market-oriented survival mode have been seriously divided, some medical institutions have fallen into a state of disorderly competition, and some pharmaceutical industry executives rely on information asymmetry, industry technology monopoly, and inflated drug prices to make profits, further aggravating the trend of abnormally high medical costs and waste of medical resources (39).

Besides, the limited overall public health resource allocation and the policy of maximizing efficiency have led to the emergence of a tiered hospital system. In many countries, hospitals are graded based on the severity, urgency, and complexity of diseases. Different levels of medical institutions are responsible for the treatment of different diseases. People typically seek medical care at local lower-level hospitals, and only escalate to higher-level hospitals if they find their health issues cannot be resolved at the lower level (40). This grading system is conducive to overall efficiency, with a large number of general diseases being treated by doctors in general hospitals, and more competent hospitals and doctors dealing with more difficult diseases. However, the problem is that many Grade A hospitals produced by this system are concentrated in some developed or more populated cities, whereas less developed cities. Although this ensures efficiency in the case of limited input, it affects equity (41). This not only makes residents in some areas face greater pressure on health services, but also aggravates the gap in medical resource allocation between developed and underdeveloped cities, urban and rural areas. Patients in less developed areas who are unsure of their condition and want a definitive diagnosis or treatment must be referred to a higher-level hospital and have to spend a lot of time and energy traveling to developed areas (42). The regional unequal allocation may also lead to patients facing higher transportation and time costs when seeking health services, which in turn deepens the medical dilemma of socially and economically disadvantaged groups (43).

### 2.2 Urban development and allocation of health resources

In the period of rapid development of each country, people tend to focus on the developed cities with large populations, ignoring other regions. However, the *per capita* disease burden and the need for health care are actually higher in places far from major population centers (44). The lack of attention to other urban and rural areas has resulted in unequal allocation of public health resources (e.g., number of doctors, number of beds, etc.) (45). However, the moderate and equitable distribution of basic health resources throughout the population is an important basis for a country’s social safety net and a necessary condition for maintaining a country’s social stability. On the whole, when the distribution of social and economic resources inevitably tends to be polarized, the equitable distribution of basic medical and health resources becomes more important for maintaining social stability. In addition, from the individual point of view, the adequacy, publicity, and convenience of healthcare services also have a significant impact on residents’ happiness in life (46).

In recent years, various countries have introduced many policies to deal with this problem, and the fairness of public health resource allocation has been improved in some countries, but it is still serious (47). In response to this challenge, it can be alleviated through cooperation mechanisms between different cities, including sharing health resources, so as to promote better coverage of health services in different cities, thereby improving the overall level of public health services (48). At the same time, some medical networks can improve the efficiency of referral by quickly sharing the information of referred patients, so as to reduce the damage to equity caused by referral problems caused by hospital classification (49). On the other hand, starting from the population level, paying attention to the small cities that are often ignored is also an effective way to promote the fair allocation of medical resources (50). By providing more health resource support to these cities, more accessible health services can be achieved, making the overall distribution of health resources more equitable.

Cities in developing countries have mostly adopted policies of rapid expansion over the past few decades, which has led to urban land sprawl and real estate booms. However, many small cities have not achieved the expected population and economic growth, but have experienced population decline and urban contraction (51). City shrinkage is fundamentally defined by population loss. Other factors include insufficient endogenous dynamism due to industrial decline and a serious homogenization of population structure. All of these can be considered as the disappearance of urban characteristics (52). The shrinking of these less developed cities is not the result of economic decline in the general sense, but due to the imbalance of regional development in the country in the past decades (53), which may lead to the spatial mismatch of “population and public resources” (54). It is unreasonable for local governments in shrinking cities to reduce the allocation of public health services in shrinking cities, which will further aggravate the problem of health equity. On the contrary, the improvement of medical quality can also reverse and promote the economic growth of cities, so more attention should be paid to the quality of health resources provision (55). In this way, the polarization of urban public health resources between different cities can be reduced (56).

Furthermore, the equity allocation of health service resources is closely related to factors such as government decision-making and talent cultivation. For example, some city leaders do not understand the allocation of medical resources, which leads to bad phenomena. City leaders have an insufficient understanding of the allocation and distribution of medical resources and often rely on experience in practice, which may lead to inaccurate and incomplete decision-making (57). This can take the form of oversupply in some regions and scarcity in others, increasing inequality in health care. In addition, there is a mismatch between the academic content of students within inner-city universities and the practical applications offered by public health professionals in the workplace. This may stem from a disconnect between academic programs and actual health service needs, a mismatch that can manifest in a lack of practical skills, a misperception of health service needs, and a lack of in-depth understanding of public health challenges that make it difficult for students to adapt to the actual work environment after graduation (58). Other factors that affect the equity of health services are the different utilization rates of health resources in cities in different regions. Different types of cities, such as tourist cities and industrial cities, have significant differences in demand and utilization of medical resources due to their special economic, social, and demographic structures, which may affect the service results for residents (59).

## 3 Data and method

### 3.1 Data resource

The data set utilized for this study was obtained from the *China City Statistical Yearbook*, covering 2010 to 2020. We extracted the number of hospital beds and doctors as dependent variables representing health services. The number of hospital beds refers to available beds in each city in different years.

**Figure 1:**
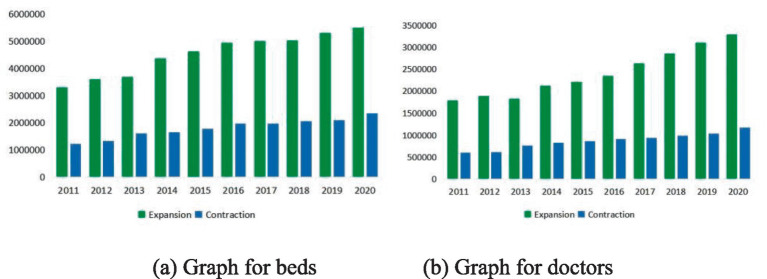
Delivery of health services in expansion and contraction cities.

The independent variable in this study was the population change rate, calculated using 2010 as the base year. Data from 2020 were chosen because the data source was China’s Sixth National Population Census. If a city’s annual population growth rate is negative, it indicates city contraction compared to 2010. This paper used a binary variable to record this, with dummy variables 1 and 0 representing city contraction and expansion, respectively. This paper employed a breakpoint regression approach, using a 0-population change rate (indicating the city is neither contracting nor expanding) as the breakpoint to analyze the situation when the number of city-provided health services is contracting or expanding. Figure 1 presents a dual bar chart illustrating health services delivery in expanding and contracting cities from 2011 to 2020. The figure comprises two sub-figures: (a) represents the number of beds, and (b) represents the number of doctors.

This study used several control variables to account for potential influences, including *per capita* GDP, financial autonomy, and the delivery of other public services, such as the number of primary schools and full-time (FT) teachers in primary schools. Previous research has shown that economic development and financial autonomy will affect the delivery of public services by local governments (60). In addition, government concerns heavily subsidize health services and education (8). Table 1 provides descriptive statistics summarizing the sample’s essential characteristics. However, there was missing data for the *per capita* gross domestic product (*per capita* GDP) in 2017, and the data for other years were complete. A linear interpolation method was employed to estimate the missing *per capita* GDP value for 2017.

**Table 1:** Descriptive statistics.

Variable	Mean	SD	Min	Max
Number of hospital beds	20352.26	18033.23	1,352	177,410
Number of doctors	10515.92	10371.85	777	118,541
Population (million)	4.46	3.41	0.23	31.98
GDP *per capita*	47392.49	33760.66	1227.6	256,877
Financial autonomy	0.45	0.22	0.06	1.54
Primary schools	612.98	548.74	18	5,248
FT teachers in primary schools	18118.12	13105.14	786	130,610

Sample size 2,739.

### 3.2 Empirical model

The study employed a regression discontinuity (RD) approach to analyze the causal relationship between city shrinkage and health services delivery. The regression discontinuity method applies to this study and can effectively identify and handle causal relationships. RD allocates interventions by utilizing a clear threshold (in this study, the point where urban population change equals 0), thereby reducing selection bias and improving internal effectiveness. This approach treated the number of health services provided as the dependent variable and the population change rate as the independent variable. Some control variables were incorporated to account for potential confounding factors and construct a robust regression discontinuity model:


(1) $$\begin{array}{l} H_{\mathrm{it}}=\alpha +\beta \left(x_{\mathrm{it}}-c\right)+\delta D_{\mathrm{it}}+\gamma \left(x_{\mathrm{it}}-c\right)\\ D_{\mathrm{it}}+\mathrm{\pi contro}l_{\mathrm{it}}+\lambda_{i}+\tau_{t}+\varepsilon_{\mathrm{it}} \end{array}$$

In Eq. 1, i denotes a province, and t denotes a year; $H_{\mathrm{it}}$ denotes the number of health services provided, including the number of hospital beds and the number of doctors; The coefficient β denotes the estimated effect of the population change rate; $x_{\mathrm{it}}$ denotes population change rate; $x_{\mathrm{it}}-c$ denotes a normalisation of $x_{\mathrm{it}}$ , meaning the breakpoint of $x_{\mathrm{it}}-c$ is 0. $\delta D_{\mathrm{it}}$ denotes processing state; $\left(x_{\mathrm{it}}-c\right)D_{\mathrm{it}}$ denotes introduced as the interaction term, meaning the slopes on each side of the breakpoint can differ. $\mathrm{contro}l_{\mathrm{it}}$ denotes control variables, $\lambda_{i}$ denotes the city fixed effect, $\tau_{t}$ denotes the year, and $\varepsilon_{\mathrm{it}}$ denotes the independent random error term. Then, we utilized the fixed effects model to comprehensively analyze the various factors that influence health services delivery:


(2) $$H_{\mathrm{it}}=\alpha +\sum \beta_{itk}x_{itk}+\lambda_{i}+\tau_{t}+\varepsilon_{\mathrm{it}}$$

In Eq. 2, $H_{\mathrm{it}}$ also denotes the number of health services delivered; k donates different independent variables x , including GDP *per capita*, financial autonomy, value added by tertiary industry, primary schools, FT teachers in primary schools; $\lambda_{i}$ and $\tau_{t}$ denote the province and the year fixed effect; $\varepsilon_{\mathrm{it}}$ denotes the independent random error term.

In this study, we use the Stata 17.0 to analyze the data. Stata is a data analysis and statistical software widely used in economics, sociology, and other fields. To determine the suitability of the regression discontinuity model, as shown in Appendices A,B, we tested the sample distribution and fitting of the model. Meanwhile, we also conducted a sensitivity analysis on the model, as shown in Appendix C. From the results, the model meets the requirements of regression discontinuity.

## 4 Results

### 4.1 Health services in city shrinkage

Table 2 reveals the impact of the population growth rate on health services, specifically the number of beds and doctors. Both regression models exhibited statistically significant breakpoints, suggesting city shrinkage potentially exerted a causal effect on health services provision. We found from the results that city shrinkage has a causal relationship with both the number of hospital beds (−1,200.4131, *p* < 0.05) and doctors (−540.0454, *p* < 0.05). A negative coefficient might indicate that contracting cities have larger disadvantaged populations and need more health resources; alternatively, it might indicate that local governments have wasted resources by forcing the delivery of too many public services to reduce the rate of city contraction. Some expanding cities may face run-on health resources, as the economy and population of an expanding city will increase before available health services do. Another possibility is that a relatively low delivery of health services limits an expanding city’s continued expansion, resulting in a lower-than-expected population growth rate.

**Table 2:** Regression discontinuity analysis of health service provision.

	Model 1		Model 2	
Variables	Number of hospital beds		Number of doctors	
Population change rate	−1409.322**	(558.1896)	−642.0145***	(250.0724)
Control variables	Yes		Yes	
Fix effects	Yes		Yes	
Observations	2,736		2,739	

Standard errors in parentheses *** *p* < 0.01, ** *p* < 0.05, * *p* < 0.1.

**Figure 2:**
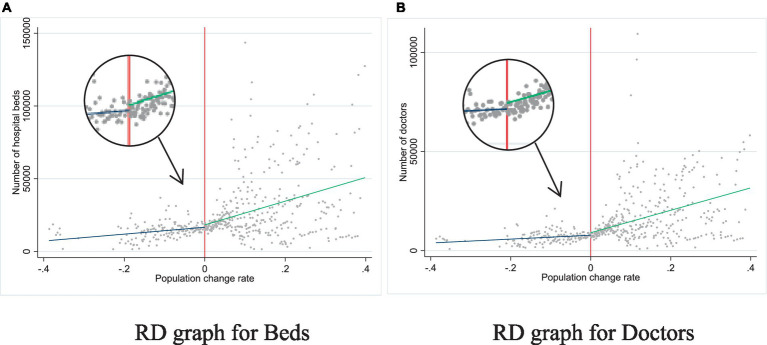
The effect of city shrinkage on the number of health services provided by local government.

Figure 2 showcases RD graphs corresponding to the two models. To the left of the red line, the graph illustrates the number of health services provided by local governments during city contraction, with the corresponding fitting line in blue. To the right, the graph portrays the number of health services provided by local governments during city expansion, with the corresponding fitting line in green. The blue and green lines are visibly divergent on both sides of the red line.

Notably, the blue line exhibits a relatively flatter slope, while the green appears steeper. This indicates that when cities contract, local government’s delivery of health services remains relatively stable, despite a substantial population decline. Initially, expanding cities might be unable to keep up with the expansion rate. However, as expansion increases, local government services in many cities change significantly. These observations suggest that the impact of population change on health services delivery differs depending on the city’s population trend (expansion or contraction). During city shrinkage, local governments may be less willing to invest in health service resources.

### 4.2 Influential factors in health service provision

Table 3 displays the effects of various variables on providing two types of health services. All variables demonstrate statistical significance. A positive *per capita* GDP coefficient (beds: 0.0782, *p* < 0.001; doctors: 0.0649, *p* < 0.001) indicates that cities with better economic development are more capable of investing in health services. Similarly, a positive financial autonomy coefficient suggests that cities with a higher government revenue-to-expenditure ratio (and, consequently, more disposable income) have a greater capacity to allocate resources to health services. Although local government financial autonomy is beneficial for the rational allocation of material resources (2,881.7673, *p* < 0.05), its impact on human resource allocation is limited. In addition, the coefficient for the number of primary schools was negative; this can be attributed to the significant financial expenditure required to construct public primary schools. When governments invest heavily in school construction, resources for health services might be impacted. This also illustrates that government investment in other public services also affects the allocation of health service resources.

**Table 3:** Regression analysis with fixed effects model on control variables.

	Model 3		Model 4	
Variables	Number of hospital beds		Number of doctors	
GDP *per capita*	0.0782***	(0.0086)	0.0649***	(0.0050)
Financial autonomy	2,881.7673**	(1,260.6347)	902.3542	(739.3160)
Primary Schools	−8.8162***	(0.5636)	−5.3949***	(0.3305)
FT teachers in primary schools	1.3008***	(0.0235)	0.7061***	(0.0138)
Fixed effects	Yes		Yes	
Constant	−17,591.8060***	(883.8212)	−14,484.8023***	(518.1289)
Observations	2,731		2,734	
R-squared	0.766		0.757	

Standard errors in parentheses *** *p* < 0.01, ** *p* < 0.05, * *p* < 0.1.

### 4.3 Robustness test

We performed a regression analysis to assess the robustness of our model, after excluding data from cities in China with higher political status or relatively advanced economic development (i.e., Beijing, Shanghai, Chongqing, Tianjin, Guangzhou, and Shenzhen) to ensure that those cities’ specific characteristics did not solely drive our findings. By excluding them, we obtained a more diverse sample of cities, allowing us to examine the relationship between population change and health services delivery in a broader context.

In Table 4, the results of the robustness checks using the RD models reaffirm the coefficients’ significance (beds: −1,200.4131, *p* < 0.05; doctors: −540.0454, *p* < 0.05), even after excluding the six more-developed cities, strengthening our findings, and suggesting that the relationship between population change and health services delivery is consistent and robust across a broader range of cities.

**Table 4:** Robustness analysis of regression discontinuity for health service provision.

	Model 5		Model 6	
Variables	Number of hospital beds		Number of doctors	
Population change rate	−1,200.4131**	(499.8058)	−540.0454**	(243.1996)
Control variables	Yes		Yes	
Fixed effects	Yes		Yes	
Observations	2,684		2,687	

Standard errors in parentheses *** *p* < 0.01, ** *p* < 0.05, * *p* < 0.1.

## 5 Discussion

### 5.1 Statement of principal findings

This is the first study to identify that city shrinkage affects health services delivery provision. Three critical discussions can be drawn from its findings.

**Figure 3:**
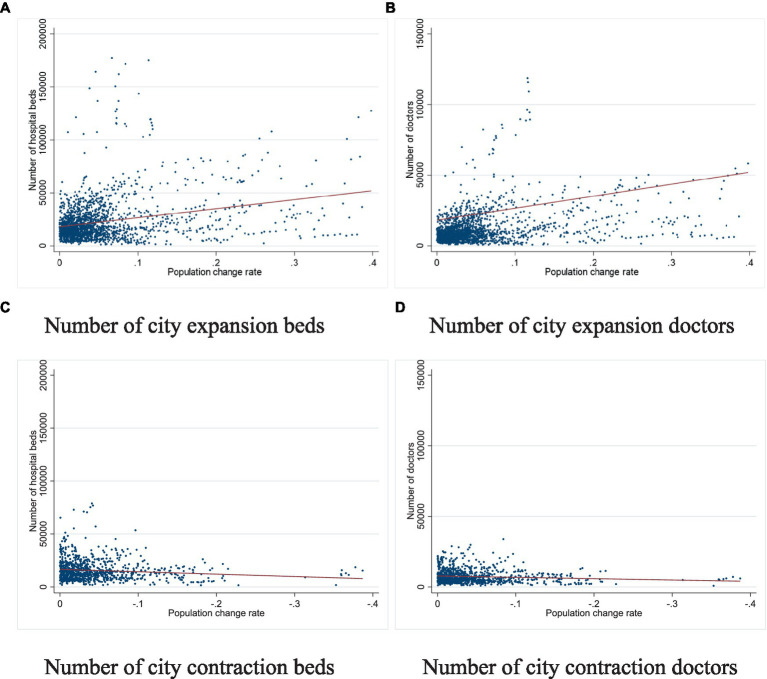
Population change rates affect health services provision.

Firstly, we find a causal relationship between city shrinkage and the allocation of health service resources. When a city experiences contraction, health service resources can significantly decrease. Specifically, there is a causal relationship between city shrinkage and the number of hospital beds and doctors. As Figure 3 shows, health service provision increases significantly in expanding cities, while contracting cities maintain a smooth curve, likely determined by policymakers’ inherent preferences in the face of population loss. From 2010 to 2020, China was in a rapid population aging phase. Older adult populations need more health services, meaning the number of primary healthcare users did not reduce as quickly as some cities’ populations. Additionally, governments must maintain the status quo for stability reasons; if too many health services are eliminated, more people will leave the city, shrinking it further and creating a vicious circle.

Second, it is important to consider endogenous factors for the growth of city health services, such as how attractive the city is to talented individuals. Most previous studies fail to comprehensively investigate health services (the number of hospitals, hospital beds, and doctors) from a socio-economic perspective, instead considering only minimal socio-economic factors. It takes work to attract talents, especially graduates and young doctors, to join hospitals in contracting cities, as many healthcare professionals might prefer to work in larger cities with more job opportunities and better pay. Although health reform is gradually deepening, hospital staff mobility is still very high. Hospitals pursue individuals with high academic qualifications and need a scientific, practical talent selection mechanism. It is impossible to fully and effectively consider whether talents’ values and personal pursuits align with the hospital, resulting in a period in which hospital talents seem vibrant. Still, it has laid the groundwork for talent loss in the long run. However, most expanding cities need more health services, so more doctors stay in hospitals and are given few opportunities, leading to a sense of not belonging to the organization and creating a vicious cycle.

Third, there is the issue of patient equity—i.e., whether health services are fairly distributed to all patients regardless of the type of city or demographic change in the context of urban contraction and expansion. On the one hand, we are very concerned about patient equity in shrinking cities. As the population of a shrinking city decreases, patients face reduced delivery of health resources. On the other hand, we also find that expanding cities may face issues such as resource constraints and extended queue times, which can affect health equity. Some studies illustrate that Chinese patients have problems with overcrowding and long waiting times for medical treatment (61). The decrease in population has led to a decrease in the willingness of the government to invest in public services, which has also affected medical services. The city shrinkage essentially damages the benefits of residents’ health services, laying hidden dangers for health services equity. Therefore, intersectional equity is therefore key to realizing the equity of health service provision under city shrinkage. This intersectional equity is reflected in human resources, financial resources, etc. (62). The government needs to pay attention to the impact of population changes.

### 5.2 Strengths and limitations

The study’s results show that the government prefers reducing health services delivery in contracting cities and increasing health services delivery in expanding cities. In our fixed-effects model, decreases in health services correlated with reductions in other public services, meaning city policymakers base public services investment decisions on population. When the population decreases, they scale back their investment in health and other public services or maintain the current level. This may be because population decline decreases demand for health services, reducing the incentive to invest in health services. At the same time, a declining population also leads to decreased fiscal revenues, so governments prioritize the efficient use of budgetary resources to meet the remaining population’s needs for essential healthcare services.

We note three limitations. First, we need to further explore the mechanisms and pathways of city contraction and expansion in health services delivery. This study identifies macro correlations through quantitative analysis but does not delve into their specific underlying mechanisms. For example, the reasons why health services remain stable during city contraction might involve government policies, community organization, and resource allocation. Second, some data were missing due to changes in the statistical methods used by *the China City Statistical Yearbook*, so interpolation was used, meaning the results might deviate slightly from reality. Third, this study lacks an examination of individual health service needs. Against the backdrop of city shrinkage, further research is needed to explore the situation of individuals accessing services. Research needs to obtain citizens’ perceptions of health services to understand whether it affects the health service equity of individuals. This is also the focus of future research.

### 5.3 Interpretation within the context of the broader literature

Existing studies on city shrinkage have mainly focused on developed countries; more research is needed on how city areas in developing countries cope with population changes and provide health resources. Considering the economic, cultural, and social environmental differences across countries and regions, in-depth research on city areas in developing countries is needed to fully understand the allocation methods and influencing factors of health service in city shrinkage contexts. Some developing countries prioritize the accessibility of primary medical services, often emphasizing fundamental needs while overlooking quality issues (63), perhaps due to challenges that pressure these countries’ fragile healthcare systems—including rapid urbanization, environmental degradation, and unfair global trade conditions (64).

As a unique, developing country, China experiences rapid expansion and ongoing shrinkage in different cities. Therefore, research within the Chinese context is fundamental. As a resource-limited developing country with a large population, China faces challenges such as increasing population, aging, and (more recently) epidemics. Health resource delivery is receiving increased government attention, and investments in various healthcare projects are rising. However, it is essential to acknowledge that health resources are inherently limited. China has undergone several reforms in health services to address the challenges related to healthcare resources, ultimately agreeing with the government’s dominant role in health services. The government must be more critical in health services (65), especially given that recent scholarship proves that medical marketization is unreasonable for most developing countries (64–66).

### 5.4 Implications for policy, practice, and research

Our study offers some implications for health services and policy designs for equity in health services provision. Governments and policymakers need to take appropriate measures to ensure patient equity: A sound planning and policy mechanism should be established to allocate healthcare resources and balance supply and demand reasonably. This includes developing comprehensive healthcare planning, anticipating the impact of demographic changes on healthcare demand in advance, and adjusting the resource allocation accordingly.

We should focus on the delivery of medical services in shrinking cities. The decrease in population may reduce the government’s willingness to invest in health resources. This may be a key factor contributing to the unfairness of medical services. Therefore, it is necessary to ensure that government investment in medical services is not affected by demographic factors.

Monitoring and evaluation mechanisms for healthcare services need to be strengthened to identify and correct inequities promptly and protect patients’ equal rights and interests in the event of urban contraction and expansion.

## 6 Conclusion

Our findings highlight the challenges that city shrinkage poses to health service provision in China. The consequences of city shrinkage for health service provision are complex and multifaceted, impacting access to healthcare opportunities for residents. Specifically, on the one hand, a causal relationship exists between city shrinkage (expansion) and the delivery of health service resources. On the one hand, there is a significant gap in the delivery of health service resources between shrinking and expanding cities. The changes in the urban population have led to an imbalance in resource allocation. On the other hand, patients confront challenges due to limited access to health services. Most studies have confirmed that the expansion of cities intensifies competition for health service resources. But in shrinking cities, the government lacks the willingness to supply resources, resulting in stagnant development of health services. In conclusion, addressing the issues of shrinkage and resource inequities demands targeted policies and programs that prioritize the spatial distribution of health resources to promote regional development and improve health outcomes.

## Data availability statement

The original contributions presented in the study are included in the article/Supplementary material, further inquiries can be directed to the corresponding author.

## Author contributions

HW: Writing – review & editing. KX: Writing – original draft, Writing – review & editing. HF: Writing – original draft. HL: Writing – original draft. HZ: Writing – review & editing.
