# Supplementary material for: Will urban scale affect health services inequity? The empirical evidence from cities in China
Source: Front Public Health. 2024 Jul 8;12:1330921. doi: 10.3389/fpubh.2024.1330921 (PMC11260790; doi:10.3389/fpubh.2024.1330921)
Supplement: Supplementary file 1 [file Data_Sheet_1.docx]

# Appendix


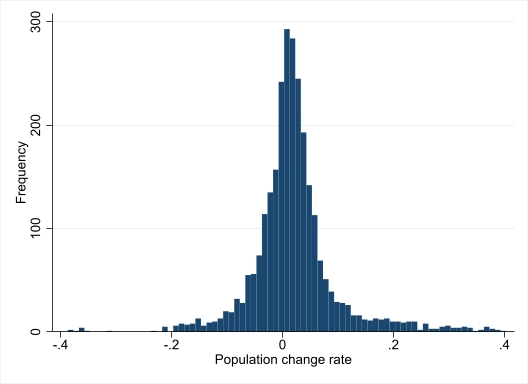


**Appendix A Feasibility testing**

**Appendix B Optimal model order determination**

| Variables | P=1 | P=2 | P=3 | P=4 |
| --- | --- | --- | --- | --- |
| Beds: AIC | 61098.0696 | 61083.5210 | 61068.1248 | 61065.7454 |
| Beds: BIC | 61121.7266 | 61119.0065 | 61115.4388 | 61124.8879 |
| Doctors: AIC | 58015.2419 | 57975.6661 | 57962.7879 | 57958.9054 |
| Doctors: BIC | 58038.9033 | 58011.1582 | 58010.1107 | 58018.0589 |

**Appendix C** **Sensitivity analysis**

| Control variables | Coefficient |
| --- | --- |
| GDP per capita | -225.3377 |
| Financial autonomy | -0.0068583 |
| Fixed effects | 5.650864 |
| FT teachers in primary schools | -483.1447 |

Note: Standard errors in parentheses *** p<0.01, ** p<0.05, * p<0.1
